# Different penalty methods for assessing interval from first to successful insemination in Japanese Black heifers

**DOI:** 10.5713/ajas.18.0733

**Published:** 2019-02-07

**Authors:** Asep Setiaji, Takuro Oikawa

**Affiliations:** 1 United Graduate School of Agricultural Sciences, Kagoshima University, Kagoshima 890-0065, Japan; 2 Faculty of Agriculture, University of the Ryukyus, Nishihara, Okinawa 903-0213, Japan; 3 Department of Animal Science, Faculty of Animal and Agricultural Sciences, Diponegoro University, Tembalang Campus, Semarang 50275, Central Java, Indonesia

**Keywords:** Interval First to Successful Insemination, Japanese Black Heifer, Penalty Method, Bootstrap, Heritability

## Abstract

**Objective:**

The objective of this study was to determine the best approach for handling missing records of first to successful insemination (FS) in Japanese Black heifers.

**Methods:**

Of a total of 2,367 records of heifers born between 2003 and 2015 used, 206 (8.7%) of open heifers were missing. Four penalty methods based on the number of inseminations were set as follows: C1, FS average according to the number of inseminations; C2, constant number of days, 359; C3, maximum number of FS days to each insemination; and C4, average of FS at the last insemination and FS of C2. C5 was generated by adding a constant number (21 d) to the highest number of FS days in each contemporary group. The bootstrap method was used to compare among the 5 methods in terms of bias, mean squared error (MSE) and coefficient of correlation between estimated breeding value (EBV) of non-censored data and censored data. Three percentages (5%, 10%, and 15%) were investigated using the random censoring scheme. The univariate animal model was used to conduct genetic analysis.

**Results:**

Heritability of FS in non-censored data was 0.012±0.016, slightly lower than the average estimate from the five penalty methods. C1, C2, and C3 showed lower standard errors of estimated heritability but demonstrated inconsistent results for different percentages of missing records. C4 showed moderate standard errors but more stable ones for all percentages of the missing records, whereas C5 showed the highest standard errors compared with non-censored data. The MSE in C4 heritability was 0.633×10^−4^, 0.879×10^−4^, 0.876×10^−4^ and 0.866 ×10^−4^ for 5%, 8.7%, 10%, and 15%, respectively, of the missing records. Thus, C4 showed the lowest and the most stable MSE of heritability; the coefficient of correlation for EBV was 0.88; 0.93 and 0.90 for heifer, sire and dam, respectively.

**Conclusion:**

C4 demonstrated the highest positive correlation with the non-censored data set and was consistent within different percentages of the missing records. We concluded that C4 was the best penalty method for missing records due to the stable value of estimated parameters and the highest coefficient of correlation.

## INTRODUCTION

Reproduction traits are important components from various breeding aspects, which have a large bearing on production and profitability. As in other livestock species, reproductive traits of beef cattle tend to be of low heritability [1]. Reproductive traits of heifers are measured relatively early in their productive life and have positive genetic correlations with reproductive and yield traits in dairy cows [2]. Thus genetic analysis of reproductive traits in heifers should be conducive to the improvement of reproductive performance without loss of genetic progress in yield traits.

The interval from first to successful insemination (FS) is the number of days between the first insemination and the insemination that results in conception. The FS in heifers has moderate heritability and high genetic correlation with the reproductive performance of cows, especially with FS and days open [3]. A problem in evaluating FS of heifers is the high number of unsuccessful inseminations termed “open heifer”. Farmers tend to cull heifers that do not conceive after a series of inseminations in that early culling can reduce the cost of feeding the animals. In the present study, heifers that did not conceive were culled and categorized under “missing FS record”. Thus, records of all animals are crucial for valid genetic analysis.

For solving the problem of missing records, several penalty methods have been proposed by animal geneticists: Adding 21 days to the largest record within a contemporary group, has been proposed based on the estrous cycle of female cattle [4]. Adding a constant number of 30, 60, or 90 days to a missing record, has been proposed, based on the number of months after the last insemination [5]. In this study, we set up one approach based on the number of inseminations and another based on the estrous cycle of female cattle. The objective of this study is to determine the best approach for handling missing records of heifer reproductive traits, for estimating the genetic parameters and for predicting the breeding value of FS in Japanese Black heifers.

## MATERIAL AND METHODS

### Data set

Reproduction records of Japanese Black heifers were obtained from Artificial Insemination Center of Northern Okinawa. A data set consisted of records of artificial insemination, calving events and FS in heifers. The data set was edited by the following requirement: heifers born between 2003 and 2015, first insemination of heifers between 2005 and 2016, and farms with a minimum of five records. The final data set comprised 2,367 records of heifers from 164 farms, including 206 (8.7% of the total) missing records. The data structure is presented in (Table 1). The FS is computed as the interval in days between the first insemination date and the last insemination date that resulted in conception. Three percentages (5%, 10%, and 15%) of the records were investigated with the use of a random censoring scheme.

### Penalty method

Two penalty approaches were used in this study: i) based on the number of inseminations, ii) based on the estrous cycle of female cattle. The FS days tend to be prolonged with the increasing number of inseminations, and based on this relationship, four penalty methods, coded C1, C2, C3, and C4, were set up. The last penalty method (C5) was based on the estrous cycle of female cattle [4]. When P is days of penalty, the methods are:

- C1: average FS according to the number of inseminations. P = n_x_, where n_x_ is the average number of FS days at the number of times till the last insemination plus one.
- C2: constant number of days (359), derived from the highest expectation of FS in the records, P = n_m_, where n_m_ = 359.
- C3: maximum number of FS days to each insemination. P = n_n_, where n_n_ is the maximum number of FS days to the nth insemination.
- C4: average of n_x_ and n_m_, where n_x_ is the average number of FS days at the number of times till the last insemination.
- C5: a constant number (21 d) was added to the highest number of FS days in each contemporary group. P = n_g_+ 21, where n_g_ is the maximum number of FS in each contemporary group.

### Statistical analysis

The general linear model (GLM) procedure of SAS 9.3 software [6] was used for preliminary analysis to test the significance of environment effects. The linear model used for FS was as follows:

$$y_{ijklmn}=F_{i}+Y_{j}+M_{k}+T_{l}+A_{m}+e_{ijklmn}$$

where *y_ijklmn_* is the observation of FS, *F_i_* the *i*th fixed effect of farm, *Y_j_* the *j*th fixed effect of year of insemination, *M_k_* the *k*th fixed effect of month of insemination, *T_l_* the lth fixed effect of artificial insemination (AI) technicians, *A_m_* the *m*th fixed effect of age class of heifers and *e_ijklmn_* the random residual of *y_ijklmn_*.

Genetic parameters were estimated using the univariate animal model by restricted maximum likelihood; estimated breeding value (EBV) for animals was predicted by the BLUP method using Asreml software [7]. Statistics for the mixed model analysis is

y=Xb+Gu+e,

where, **y** = a vector of observation, **b** = a vector of fixed effect of farm and AI technician, **X** = an incidence matrix for the fixed effects, **u** = a vector of random genetic additive effect, **Z** = an incidence matrix for the random effect, and **e** = a vector of random residuals.

The expectations for **y**, **u**, and **e** are

$$E\;\left[\begin{array}{c} y\\ u\\ e \end{array}\right]=\left[\begin{array}{c} \mathrm{Xb}\\ 0\\ 0 \end{array}\right]$$

The variance-covariance structure of random effects is

$$V\;\left[\begin{array}{c} u\\ e \end{array}\right]=\left[\begin{array}{cc} G & 0\\ 0 & R \end{array}\right]$$

and $G=A\sigma_{a}^{2},R=I\sigma_{e}^{2}$, where ***A*** is the numerator relation matrix, ***I*** the identity matrix $\sigma_{a}^{2}$ and $\sigma_{e}^{2}$ the additive genetic variance and residual variance, respectively.

### Bootstrap method

The bootstrap method is one of the most powerful variance estimation techniques applied to complex sample statistics, whereby simulation is conducted to generate multiple data sets from an original data set [8]. The principle of bootstrap is based on resampling of records from a current data set, that is composed of pseudo samples distributed according to the same distribution as of the original sample. A data set with missing values is generated by a random sampling scheme from the original data by replacement. Although each resampled data set has the same number of observations as the original sample, the composition of the data set is different. Therefore, each of these data sets randomly deviates from the original data set.

In the present study, the bootstrap method was used to compare five penalty methods in terms of bias, mean squared error (MSE) and the accuracy of EBV. Resampling was generated 100 times from the original data. The bias and error variance computation requires between 50 and 200 resamplings [9]. The number of bootstrap replications suggested is a minimum of 100 for standard error estimation [10]. Nonetheless, an approach using the bootstrap technique has been proposed for obtaining robust estimates of heritability [11] and [12]. In the present study, the steps of bootstrap were as follows:

- Generate a resampled data set 100 times from the original (non-censored) data.
- Choose 8.7% of missing records within each resampling data set by the random censoring scheme.
- Estimate genetic parameters and predict EBV for each data set.
- Calculate the average of genetic parameters and predicted EBV.
- Compare the penalty methods by bias and MSE of heritability and correlation of EBV with a non-censored data set.
- Repeat steps 1 to 5 for three other percentages (5%, 10%, and 15%) to assess the influence of different proportions of missing records on the inference.

## RESULTS

Results of GLM of non-genetic effects for FS with the use of non-censored data are presented in Table 2. The fixed model analysis showed that the effects of farm and the AI technician were statistically significant, while other factors did not show any significant effect. Accordingly, only the farm and the AI technician were included in the model for estimation and prediction. Table 3 show the basic statistics of FS in the penalty methods at each percentage of the missing records. The mean FS in C5 was higher than in the other methods. On the other hand, among the methods based on the number of inseminations, the highest and the lowest mean FS was observed in C2 and C3, respectively.

Additive genetic variances, error variances and heritability for non-censored data and five different penalty methods are shown in Table 4. The estimated genetic variance, residual variance and heritability of the non-censored data set were 136.15; 10,527; 0.012, respectively. C1 showed high heritability ranging between 0.011±0.019 and 0.014±0.016. The lowest heritability was 0.009±0.017 and the highest was 0.015±0.019 in C2. C3 also showed heritability ranging between 0.008± 0.019 and 0.015±0.019. C4 demonstrated medium heritability, whereas C5 showed unstable heritability. All penalty methods showed large standard errors of heritability.

Bias of estimated heritability in five penalty methods is pre sented in Table 5. C1 and C2 had bias ranging between 0.61 and 1.91 and between 0.54 and 2.71, respectively. The same trend was observed in both methods, where the lowest bias occurred in 10% and the highest in 15% of the missing records. The bias in C3 ranged between 0.79 and 2.54, with the lowest in 5% and the highest in 10%; in 8.7% it was lower than in the other penalty methods. The bias in C4 ranging between 0.48 and 2.49, demonstrated fluctuation within different percentages of the missing records. C5 showed the highest bias among the penalty methods at all percentages of missing records.

The MSE of estimated heritability in five penalty methods is presented in Table 6. The result showed that C1 had the highest MSE in 8.7%, and a variable MSE between different percentages of missing records. C2 showed a moderate MSE, ranging between 1.047 and 1.552, and the trend increased as the percentage of missing records became higher. C3 showed a low MSE, the lowest occurring in 5% and increasing as missing records increased. The highest MSE was observed at 15% of missing records. C4 showed a moderate MSE of estimated heritability, ranging between 0.633 and 0.879. C5 had a larger MSE ranging between 1.423 and 1.876.

The average correlation between the EBV of the five pen alty methods and the EBV from the non-censored data set is shown in Table 7. The correlation in C1 in 8.7% of the missing records was 0.82, 0.84, and 0.83 for heifer, sire and dam, respectively. The correlation between C1 and non-censored data was high in 5% and decreased as the percentage of missing records increased. C2 and C3 showed moderate correlation in 8.7% (0.81, 0.87, and 0.83) and (0.86, 0.90, and 0.86) for heifer, sire and dam, respectively. The highest correlation was found in C4 (0.88, 0.93, and 0.90) for heifer, sire and dam, respectively. C5 showed the lowest correlation (0.41, 0.46, and 0.41) for heifer, sire and dam, respectively, with the same trend as in C1, C2, and C3 in terms of different missing records.

## DISCUSSION

### Estimated parameters

Heifers that have a true FS are those with successful insemination, gestation and calving. Eliminating an open heifer from the data set would yield biased or underestimated genetic parameters [13]. In the present study, genetic and error variances tended to be large when the penalty data were included, and those in C4 were the lowest among the five penalty methods in 8.7%, whereas those in C5 were the highest. The effect of 30, 60, and 90 penalty days on calving day and age at first calving in the Angus heifer has demonstrated that genetic and error variance increase when a high penalty score is included in the analysis [5]. They have concluded that the smallest number of penalty days is recommended in genetic analysis because their result showed that the lowest standard error of heritability was estimated at the smallest penalty score.

In the present study, heritability of a non-censored data set was 0.012±0.016, lower than the average estimates in C1, C2, and C3, but slightly higher than those in C4 and C5. Standard errors of heritability of penalty methods were all higher than the heritability of a non-censored data set. Compared with previous reports, our heritability estimates were within the range of estimates in the literature. For Holstein heifers [3,14] and Ayrshire heifers [15], FS heritability has ranged between 0.01 and 0.02.

In the present study, all of the penalty methods showed posi tive bias in heritability estimates. The bias in C5 was higher than that in the other penalty methods, making its error variance five times higher than the other penalty methods.

The MSE is the most important criterion in evaluating the performance of a predictor or an estimator; it is also useful in acquiring the concepts of bias and accuracy in statistical estimations [16]. Estimates with a small MSE are better because they are closer to the real value [17]. Taking those aspects into consideration in the present study, MSE was calculated to compare the penalty methods: C4 showed the lowest MSE; however, C1 and C5 were both higher, demonstrating that C4 was better than the other methods. This result indicates that true FS of animal with missing records may be higher than the average number of FS days at the number of times till the last insemination but lower than the highest expectation of FS in the records. Accordingly C1 and C2 are inadequate because they are too extreme to infer true FS, whereas C4 seems to have properties that reflect true FS.

### Percentage of missing records

In the present study, 8.7% of the records were missing, and 5%, 10%, and 15% were designed to assess the influence of the missing records. The genetic and error variances in C1 and C5 tended to increase as the percentage of missing records increased; in C2, C3, and C4 they were inconsistent in the 10% and 15% missing records; in C4 they were the lowest in 5%, 8.7%, and 15% as compared with the other methods; however, in 10% of the missing records, the lowest variance was observed in C3. The reason for the fluctuation in genetic and error variances in 10% and 15% of the missing records was attributable to changes in pedigree structure. This phenomenon may be due to the deletion of key animals with regard to pedigree.

C1 showed the lowest MSE in 5% and the highest in 8.7% compared with the other percentages. In C2 MSE tended to be similar to those in C3 and C4, where the lowest MSE occurred in 5% and increased as the percentage of missing records became higher. In C4 MSE was lower than in the other methods at all percentages of missing records. C5 showed the highest and the most unstable MSE among the different percentages. When the percentage of missing records rises up to 15%, heritability tends to be lower because genetic variance decreases, while error variance increases. The change in genetic and error variances is reinforced with higher missing percentages in conjunction with changes in the data structure [18].

### Prediction of estimated breeding value of heifer and parent

The coefficient correlation of EBV for heifer was lower than that for sire and dam, whereas it was higher for sire in 8.7%, 10%, and 15% of the missing records. In 5% of the missing records, it was higher in C1 for heifer than for sire, whereas in C2 it was higher for heifer than for dam or sire. In the other percentages, it was lower for heifer than for sire or dam, which may be attributable to the decreased accuracy for heifer EBV at higher percentages of missing records.

The coefficient correlation in C3 was lower than in C4 but higher than in the other three methods. In C4 it was consistent and highest for heifers, sires and dams at all the percentages of missing records. In C5 it was the lowest for heifer and parents among the five penalty methods. The penalty in C5 was based on the highest number of FS days, as identified in each contemporary group plus the constant number (21 d). Thus the penalty by the simple addition of certain days resulted in a less accurate EBV and a lower correlation than the other methods. This phenomenon could be due to changes in EBV ranking when penalty data are implemented [19]. Smaller changes in EBV ranking may result in C4 attaining the highest correlation with a non-censored data set, demonstrating that the penalty data in C4 are the most appropriate for handling missing records of FS in genetic analysis.

## CONCLUSION

This study indicates that C4 is the best penalty method for missing records because it has the lowest MSE and the average of standard errors for heritability. It also demonstrated the highest accuracy for EBV and consistent results for all the percentages of missing records.

## Figures and Tables

**Table 1:** Structure of source data

Class	Number
Heifer with data	2,161
Open heifer	206
Sire	101
Dam	1,317
Farm	164
Age1)	3
Animal in pedigree	15,600

1) Age class: <16, 16 to 19 and >19 month.

**Table 2:** Statistical significance of non-genetic effects of first to successful insemination for non-censored data

Effect	DF	Mean square	F value	Probability
Farm	163	21136.68	2.21	0.0448
Year	11	19814.45	2.07	0.0941
Month	11	7847.10	0.82	0.6242
AI technician	5	46399.24	4.84	0.0139
Age	2	29934.07	3.12	0.0715

DF, degrees of freedom; AI, artificial insemination.

**Table 3:** Basic statistics of first to successful insemination in the penalty methods at each percentage of missing records

Method	Percentage of missing							

	5%		8.7%		10%		15%	
			
	Mean	SD	Mean	SD	Mean	SD	Mean	SD
C1	59.34	72.64	64.78	78.12	65.69	91.63	70.73	92.33
C2	72.18	82.34	78.18	86.47	80.21	87.58	93.03	95.84
C3	60.26	71.02	64.93	75.52	68.16	78.13	72.89	82.88
C4	64.83	78.36	68.45	80.29	71.87	81.43	78.15	87.52
C5	107.43	120.60	111.35	123.04	113.23	124.97	119.12	131.26

SD, standard deviation.

**Table 4:** Genetic variance, error variance and heritability of first to successful insemination for non-censored data and for each percentage of missing records in five penalty methods

Method	Percentage of missing	σ^2^_g_	σ^2^_e_	h^2^±SE
	non-censored data	136.15	10,527	0.012±0.016
C1	5%	193.76	13,808	0.014±0.016
C2		199.07	14,669	0.013±0.019
C3		172.65	13,190	0.013±0.019
C4		140.31	11,366	0.012±0.018
C5		891.07	61,925	0.015±0.023
C1	8.7%	210.95	14,654	0.014±0.020
C2		250.54	16,743	0.015±0.019
C3		197.07	13,887	0.015±0.019
C4		151.25	13,260	0.011±0.018
C5		1051.6	104,080	0.010±0.021
C1	10%	217.59	15,943	0.013±0.019
C2		224.08	18,066	0.012±0.017
C3		113.86	13,599	0.008±0.019
C4		171.63	16,469	0.010±0.018
C5		1694.5	111,500	0.015±0.021
C1	15%	231.04	20,307	0.011±0.019
C2		199.11	21,068	0.009±0.017
C3		263.13	16,743	0.008±0.019
C4		139.81	14,035	0.010±0.019
C5		2033.8	161,740	0.012±0.022

σ^2^_g_, additive genetic variance; σ^2^_e_, error variance; h^2^, heritability; SE, standard error.

**Table 5:** Bias of estimated heritability (×10^−3^) in five penalty methods

Method	Percentage of missing			

	5%	8.7%	10%	15%
C1	1.47	1.12	0.61	1.91
C2	0.64	1.17	0.54	2.71
C3	0.79	1.05	2.91	2.54
C4	0.48	2.12	0.57	2.49
C5	2.18	2.39	2.53	2.67

**Table 6:** Mean square error (×10^−4^) of estimated heritability in five penalty methods

Method	Percentage of missing			

	5%	8.7%	10%	15%
C1	0.776	1.899	1.344	1.453
C2	1.047	1.334	1.337	1.552
C3	0.786	1.266	1.345	2.781
C4	0.633	0.876	0.879	0.866
C5	1.442	1.876	1.423	1.775

**Table 7:** Average of correlation between estimated breeding value of five penalty methods and of non-censored data set in first to successful insemination

Percent of missing	Method	Heifer	Sire	Dam
5%	C1	0.89	0.87	0.90
	C2	0.87	0.85	0.86
	C3	0.92	0.94	0.92
	C4	0.92	0.95	0.93
	C5	0.50	0.60	0.52
8.7%	C1	0.82	0.84	0.83
	C2	0.81	0.87	0.83
	C3	0.86	0.90	0.86
	C4	0.88	0.93	0.90
	C5	0.41	0.46	0.41
10%	C1	0.79	0.82	0.79
	C2	0.77	0.85	0.80
	C3	0.82	0.87	0.84
	C4	0.87	0.91	0.89
	C5	0.43	0.44	0.41
15%	C1	0.69	0.71	0.73
	C2	0.71	0.81	0.73
	C3	0.73	0.81	0.76
	C4	0.78	0.87	0.83
	C5	0.39	0.42	0.38
